# Low brain endocannabinoids associated with persistent non-goal directed nighttime hyperactivity after traumatic brain injury in mice

**DOI:** 10.1038/s41598-020-71879-x

**Published:** 2020-09-10

**Authors:** Alexandra Vogel, Annett Wilken-Schmitz, Regina Hummel, Manuel Lang, Robert Gurke, Yannick Schreiber, Michael K. E. Schäfer, Irmgard Tegeder

**Affiliations:** 1 Institute of Clinical Pharmacology, Medical Faculty, Goethe-University Frankfurt, Frankfurt, Germany; 2 Department of Anesthesiology, University Medical Center, Johannes Gutenberg-University Mainz, Mainz, Germany

**Keywords:** Circadian rhythms and sleep, Cognitive neuroscience, Diseases of the nervous system, Learning and memory, Motor control, Reward, Sensory processing, Somatosensory system, Lipids

## Abstract

Traumatic brain injury (TBI) is a frequent cause of chronic headache, fatigue, insomnia, hyperactivity, memory deficits, irritability and posttraumatic stress disorder. Recent evidence suggests beneficial effects of pro-cannabinoid treatments. We assessed in mice levels of endocannabinoids in association with the occurrence and persistence of comparable *sequelae* after controlled cortical impact in mice using a set of long-term behavioral observations in IntelliCages, motor and nociception tests in two sequential cohorts of TBI/sham mice. TBI mice maintained lower body weights, and they had persistent low levels of brain ethanolamide endocannabinoids (eCBs: AEA, OEA, PEA) in perilesional and subcortical ipsilateral brain tissue (6 months), but rapidly recovered motor functions (within days), and average nociceptive responses were within normal limits, albeit with high variability, ranging from loss of thermal sensation to hypersensitivity. TBI mice showed persistent non-goal directed nighttime hyperactivity, i.e. they visited rewarding and non-rewarding operant corners with high frequency and random success. On successful visits, they made more licks than sham mice resulting in net over-licking. The lower the eCBs the stronger was the hyperactivity. In reward-based learning and reversal learning tasks, TBI mice were not inferior to sham mice, but avoidance memory was less stable. Hence, the major late behavioral TBI phenotype was non-goal directed nighttime hyperactivity and "over-licking" in association with low ipsilateral brain eCBs. The behavioral phenotype would agree with a "post-TBI hyperactivity disorder". The association with persistently low eCBs in perilesional and subcortical regions suggests that eCB deficiency contribute to the post-TBI psychopathology.

## Introduction

Traumatic brain injury (TBI) is a major cause of death and disability^1^, and survivors often suffer from persistent deficits of motor and cognitive functions, and a high incidence of chronic pain and posttraumatic stress disorder (PTSD)^2^. Even mild traumatic brain injuries often lead to persistent fatigue, unstable mood and poor concentration, insomnia, memory deficits, depression or hyperactivity and chronic pain^3,4^. Mostly, these symptoms resolve, but they persist in some patients over months regardless of the injury severity. Although early predictors of unfavorable outcomes were identified such as emotional distress and age^5^, there are no established preventive strategies, and particularly chronic post TBI pain, partially co-incident with depression or PTSD, is mostly not sufficiently responsive to psychosocial interventions or typical analgesics^6^.

Chronic pain is one of the most frequent persistent complains after TBI including headaches, chronic widespread pain and central pain^7,8^. Diffusion state magnetic resonance imaging (MRI) studies revealed white matter tract abnormalities that would agree with deficits of functional pain connectivity^9^. Alterations of BOLD signals in functional MRI studies and metabolic changes further suggested that TBI disturbs normal activity within the "pain matrix", particularly insular and prefrontal cortex^10,11^, and injuries involving supra-spinal pain inhibitory regions were associated with a high risk of post TBI pain^12^. Endogenous pain inhibition essentially requires endocannabinoid signaling^13–16^. It is not well studied if rodents like humans develop at state of central "pain" after TBI, but rodents show post-TBI anxiety^17^, which would agree with lowering of the endocannabinoid tone^18,19^.

The endogenous cannabinoid system is one of the major endogenous pain inhibitory systems. It is anti-inflammatory via the cannabinoid 2 receptor (CB2)^20^ in TBI models, and it is neuroprotective via presynaptic CB1^21–23^ by reducing glutamate excitotoxicity^22,23^ (among other mechanisms). A number of studies have demonstrated a beneficial outcome of TBI in rodents with CB2-agonists^24–26^ or inhibitors of fatty acid amide hydrolase (FAAH) that catalyzes the breakdown of ethanolamide endocannabinoids (eCBs)^27–29^. The beneficial effects of FAAH inhibitors lead to the hypothesis of "TBI-evoked eCB deficiency", which needs to be verified, and would encourage further studies with FAAH inhibitors. Astonishingly, mortality after TBI was reduced in marihuana users^30^ and a CB1/CB2 dual agonist reduced the mortality of comatose TBI patients with severe head injuries in a clinical study^31^. In addition, a recent randomized clinical MRI study showed that tetrahydrocannabinol (THC) altered threat-related processing in PTSD patients^32^, agreeing with the current view of cannabinoid-enhancing therapeutic approaches as new avenues in the treatment of PTSD^33,34^.

The majority of experimental studies using a number of different controlled brain injuries in rodents focused on the very early period after TBI, in particular the cellular, inflammatory and molecular mechanisms that orchestrate beneficial shielding of non-injured tissue and repair, but also pro-inflammatory secondary damage^35–37^. In contrast to the substantial advance in the understanding of early post-TBI events, long-lasting, protracted changes of behavior are less well studied. We found previously in the controlled cortical impact model (CCI) that TBI mice have persistent anxiety-like behavior up to 7 months after TBI^17^, which is reminiscent of the mal-processing of threat signals in human PTSD^32^. Coping styles, personality, emotion and social networks become increasingly influential for the overall long-term outcome in humans. To some extent, this may also hold true for mouse models, and these variables are influenced by the endocannabinoid tone^38,39^.

To address the complexity of a putative late "post-TBI disorder" in mice we used the CCI model of TBI and studied brain and plasma endocannabinoids in parallel with nociception and long-term behavioral analyses in IntelliCages that provide readouts of activity, circadian rhythms, "sleep", social interactions, hedonic and anhedonic learning and memory.

## Results

### Long-lasting perilesional inflammation and lowering of brain endocannabinoids

**Figure 1:**
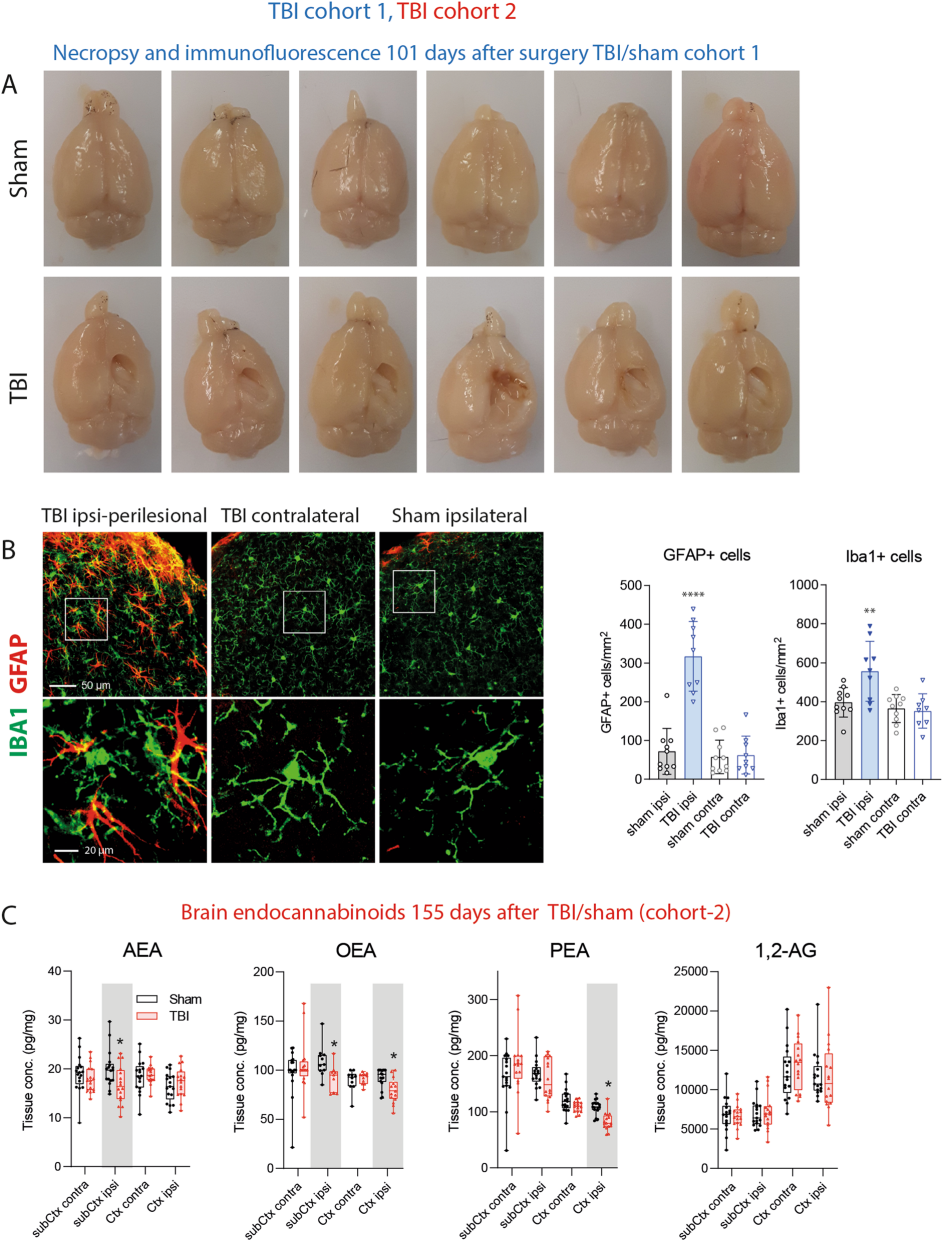
Necropsy, histology and lipids of TBI and sham mice. (**A**) Necropsy of exemplary TBI and sham mice of cohort-1, 101 days after surgery. (**B**) Immunofluorescence analysis in exemplary TBI and sham mice of cohort-1. The images show the microglial marker, ionized calcium-binding adapter molecule 1 (IBA1) and the astroglial marker, glial fibrillary acidic protein (GFAP) in the perilesional and corresponding contra-lesional/contra-lateral area. The images were captured on an LSM510 confocal microscope (Zeiss) and exported from LSM Image Browser 4.2 to Adobe Photoshop CC2018 for a global adjustment of color balance. Scale bars are 50 µm and 20 µm for upper and bottom panels. The number of IBA1 + and GFAP + cells was counted with the particle counter implemented in FIJI ImageJ (https://imagej.net/Fiji) after threshold settings according the 'auto' detection. The bars show mean and SD, the scatter show individual mice. Asterisks show significant differences versus sham ipsilateral, one-way ANOVA, posthoc Holm Šidák, adjusted ****P = 0.0001, **P 0.0091. (**C**) Box/scatter plots showing brain endocannabinoid concentrations in ipsilateral perilesional cortex (Ctx) and subcortex (subCtx) and corresponding regions of the contra lateral side 155 days after TBI or sham surgery (cohort-2). The endocannabinoids anandamide (AEA), oleoylethanolamide (OEA), palmitoylethanolamide (PEA) and 1 plus 2 arachidonoylglycerol (1,2-AG) were analyzed per tandem LC–MS/MS. The box shows the interquartile range, the line is the median, the whiskers show minimum to maximum, the scatter are individual mice. Data were compared per 2-way ANOVA ("site" × "group") and subsequent posthoc t test for the between subject factor group, i.e. TBI versus sham. Asterisks show multiplicity adjusted P values (*< 0.05). Graphs were created with Graphpad Prism 8.4 (https://www.graphpad.com), exported as enhanced metafile (emf), and arranged and labeled in Adobe Illustrator CC2020 (https://www.adobe.com/de), and exported to TIFF format.

The necropsy at 101 days after CCI revealed the extensive damage of the sensory and motor cortex (Fig. 1A, cohort-1). Immunofluorescence studies of the astrocyte marker, GFAP and the microglia marker, Iba1 showed an ipsilateral cortical astrogliosis and microgliosis (Fig. 1B; cohort-1) suggesting still ongoing inflammatory processes. Endocannabinoids are supposed to contribute to the brain's repair mechanisms after TBI by reducing hyperexcitability, vasoconstriction, edema and inflammation^21,40–42^. The analysis of key eCBs in subcortical and perilesional brain tissue 155 days after TBI (cohort-2) revealed a deficiency of the ethanolamide-eCBs, AEA, OEA and PEA, on the ipsilateral side whereas 1,2-AG (sum of 1-AG and 2-AG) was in the normal range (Fig. 1C).

AEA and 1,2-AG are agonists of the typical cannabinoid receptors, CB1 and CB2. OAE and PEA are not ligands of CB1 or CB2, but act via nuclear receptors such as PPARs, "orphan" G-protein coupled receptors such as GPR55, GPR18 and GPR119, which are also activated via AEA. Hence, AEA is the most versatile eCB. It has potent anti-vasoconstrictive properties not met by other eCBs^43^ and provides a defense against glutamate excitotoxicity^23^ and epilepsy^29,44,45^. PEA appears to exert its protective effects by decreasing the development of cerebral edema and inflammation^46,47^. It is also protective in models of epilepsy^48,49^. OEA regulates satiety, the sleep–wake cycle and has anti-nociceptive properties^50–53^. Interestingly, PTSD patients had low hair OEA concentrations^54^, and a human positron emission tomography study in PTSD patients observed low brain AEA in combination with an increased availability of free "unused" CB1 receptors^55^.

The pattern of eCB alterations at this late time after TBI in our mice suggests a weakening of the neuroprotective features of the eCB system. TBI causes cortical and subcortical network hyperexcitability, mainly originating from a loss of inhibitory control^56–58^. The observed deficiency of eCBs in the TBI-brain suggested that the disinhibition is contributed by the eCB loss. The hypothesis is supported by therapeutic effects of FAAH inhibitors in rodent TBI^27,28^. We therefore studied the associations of eCB levels with behavioral readouts, and we interpret behavioral phenotypes after TBI in light of TBI-evoked brain eCB deficiency.

### Rapid recovery of motor functions but persistence of lower body weight

**Figure 2:**
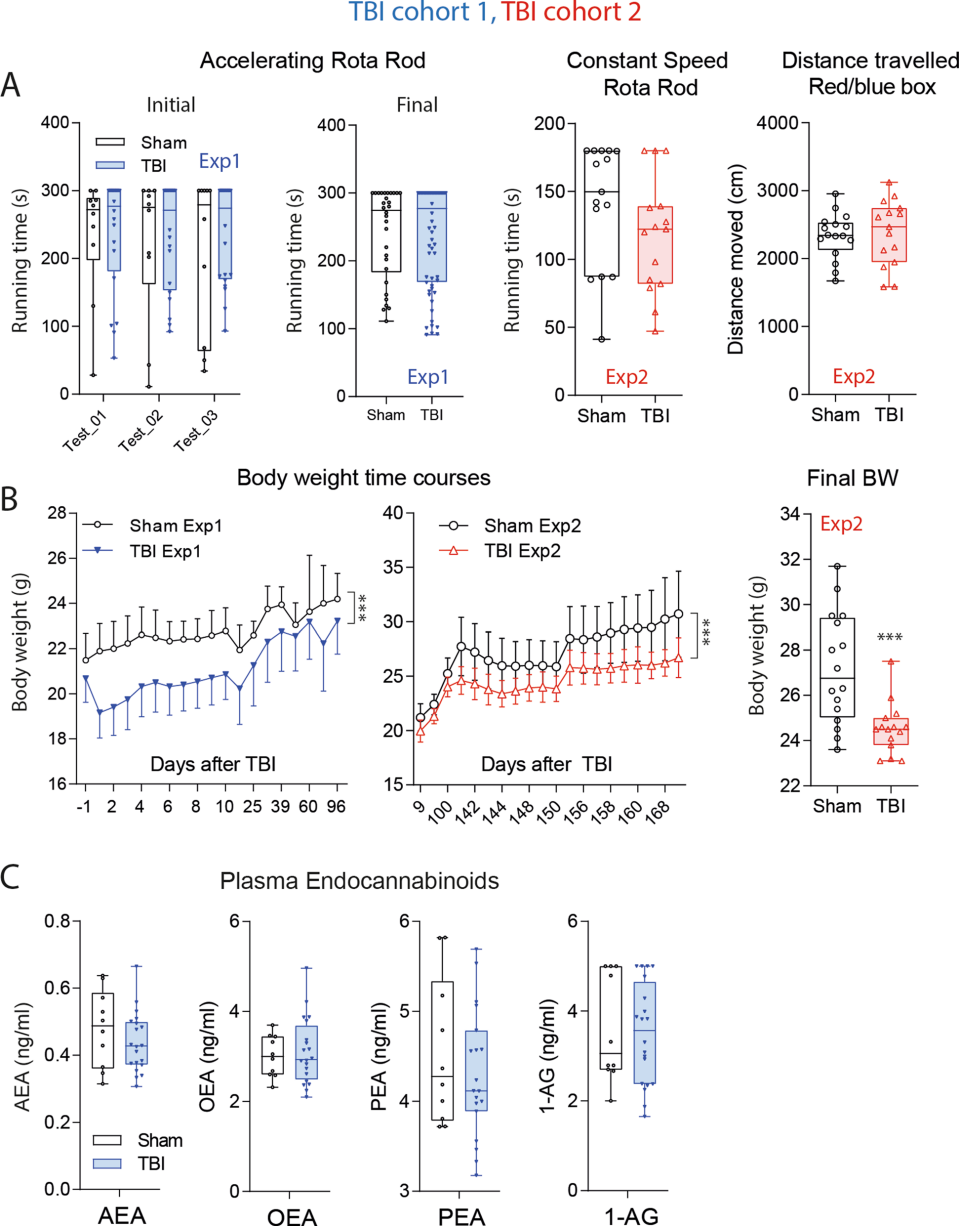
Motor functions and general well-being in TBI versus sham mice in a controlled cortical impact model. (**A**) Box/scatter plots showing running times in rotarod trials with accelerating speed or constant speed in experiment-1 with TBI/sham cohort-1 (blue) and experiment-2 with TBI/sham cohort-2 (red). Initial tests were done 10–14 days after TBI before tests of nociception and cognition. Final rotarod runs were done after completion of IntelliCage experiments 3 months (Exp1) or 6 months (Exp2) after surgery. The right panel shows the distance traveled in the red/blue box. The box shows the interquartile range, the line is the median; the whiskers show minimum to maximum, each scatter is a mouse. In 'Final-Exp1’ each mouse is represented with 2 trials. (**B**) Time courses of the body weights (means ± SD) in TBI and sham mice up to 35 days after surgery in Exp1 (blue, n = 10 sham, n = 21 TBI) and up to 170 in Exp2 (red; n = 15 both groups). Asterisks indicate significant differences between groups (2-way ANOVA for time courses, factor "group", 2-sided independent T test for comparison of final body weights, ***< 0.001; P adjusted according to Šidák for comparison of individual time points). (**C**) Plasma concentrations of endocannabinoids: anandamide AEA, oleoylethanolamide, OEA, palmitoylethanolamide, PEA and 1-arachidonoylglycerol, 1-AG in TBI/sham mice of cohort-1 at the end of the observation 101 days after surgery. The box shows the interquartile range, the line is the median; the whiskers show minimum to maximum, each scatter is a mouse. Concentrations did not differ significantly between groups. Graphs were created with Graphpad Prism 8.4 (https://www.graphpad.com), exported as emf and arranged and labeled in Adobe Illustrator CC2020 (https://www.adobe.com/de), and exported to TIFF format.

The necropsy showed the extensive cortical damage (Fig. 1A, cohort-1) but astonishingly, TBI mice were able to maintain normal running in the rotarod and travelled equal distances in the Red/Blue box 10–14 days after TBI (Fig. 2A). Rotarod running was also normal at late time points. TBI mice however, did not catch up with the controls' body weights throughout the observation up to 6 months after the injury (Fig. 2B), although they regained weight after the surgery. In contrast to eCBs in brain, plasma levels were in the normal range at this late time point (Fig. 2C), and neither plasma, nor brain eCB levels were directly associated with individual body weights (Supplementary Figure S1A,B). The weight curves suggested higher energy expenditure or reduction of feeding, but estimates of food consumption based on the weighing of food pellets were similar in both groups. Still, eCBs are key regulators of appetite and satiety and hedonic nature of pleasant feeding^59,60^, and low eCB may lead to a loss of pleasure^60^. It is of note, that hedonic sweet drinking is associated with descending pain inhibition that requires CB1 activation^16^.

### Heterogeneous nociceptive thresholds: from sensory loss to hypersensitivity

It is not well studied if rodents like humans develop a state of central nociceptive hypersensitivity or spontaneous central "pain" after TBI. Nociceptive readouts mostly rely on (paw) withdrawal or equivalent responses and depend on motor functions. The fast recovery of locomotion and motor coordination (Fig. 2A) was a prerequisite for studying nociception in this model.

**Figure 3:**
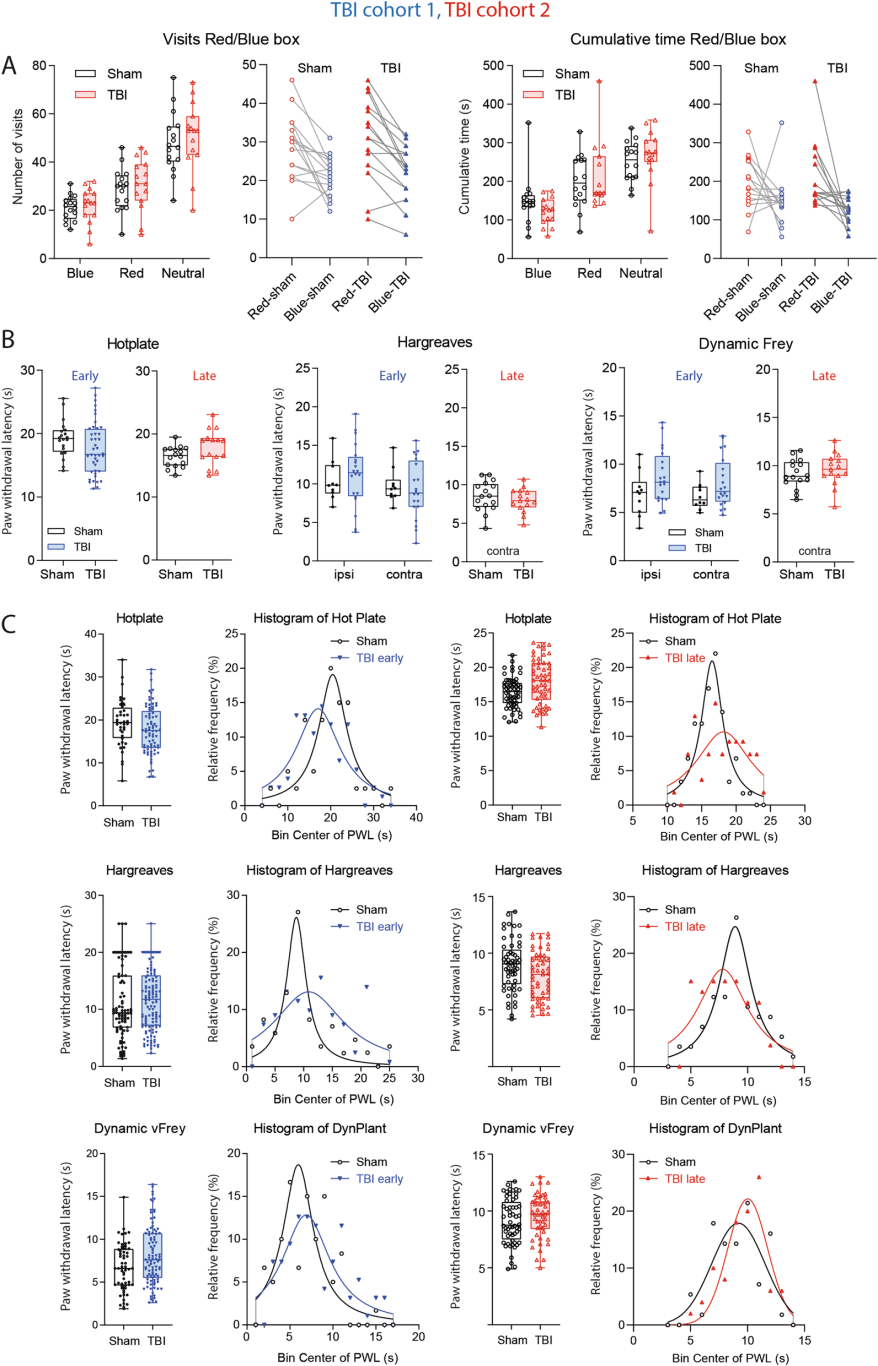
Nociceptive behavior in TBI versus sham mice. (**A**) Box/scatter plots and paired analysis of visits and cumulative times spent in red and blue chambers of a 3-compartment Red/Blue Box. The middle compartment was neutral. Both groups similarly avoided blue light. Blue light hypersensitivity is an indicator of headache. The experiments were done in TBI/sham cohort-2, 14 days after surgery. The box shows the interquartile range, the line is the median; the whiskers show minimum to maximum, each scatter is a mouse. (**B**) Box/scatter plots show nociceptive paw withdrawal responses elicited by heat or mechanical stimulation in hot plate, Hargreaves and dynamic von Frey experiments. Early tests were done 14 days after controlled cortical impact or sham surgery in TBI/sham cohort1 (blue, n = 10 sham, n = 21 TBI), late tests 5 months after surgery in TBI/sham cohort-2 (red, n = 16 sham, n = 15 TBI). The box shows the interquartile range, the line is the median; the whiskers show minimum to maximum, each scatter is a mouse. In early hot plate tests, each mouse is represented with two trials. Ipsilateral and contra lateral refer to the side of the cortical trauma, which receives input from the contra lateral paw. (**C**) Box/scatter plots of pooled data from several trials and frequency distribution of the response latencies, which were fitted to a normal distribution according to Gauss or Lorentz, depending on the goodness of fit parameters. Although mean withdrawal latencies did not differ between groups (unpaired, 2-tailed T tests), Gauss curves revealed a higher variance in TBI mice, particularly for heat stimulation. Graphs were created with Graphpad Prism 8.4 (https://www.graphpad.com), exported as emf and arranged and labeled in Adobe Illustrator CC2020 (https://www.adobe.com/de), and exported to TIFF format.

In previous studies, we found that trigeminal nerve injury increased blue light avoidance^61^ presumably owing to upregulations of melanopsin in injured nerves^62^, leading to blue light-triggered headache or migraine. However, in the present study in the Red/Blue box, both groups equally preferred the red light (Fig. 3A), and TBI mice behaved completely normal including visits and time spent in the respective chambers (Fig. 3A) suggesting that they had no trigeminal hypersensitivity. The result agrees with previous studies where "TBI-headache" was only measurable after injection of nitroglycerin or facial stimulation^63,64^ or after repetitive head injuries^65^.

The brain homunculus suggests that the CCI model primarily leads to a damage of the cortical representation of the lower limb and paw. Therefore, we assessed paw withdrawal latencies upon heat or mechanical stimulation (Fig. 3B,C). The average withdrawal latencies did not significantly differ between groups irrespective of the test and stimulus, and ipsilateral and contralateral latencies were similar. The contralateral paw is projecting to the side of the cortical injury. Analysis of the distribution of nociceptive responses using pooled data of daily measurements showed that the Gauss distributions were flattened in TBI mice (Fig. 3C) showing an increase of variability with a response spectrum ranging from loss of sensitivity to hypersensitivity. The effect only partially resolved over time (evident at 10–14 days and 5–6 months), and was stronger for heat than mechanical stimuli (Fig. 3C). Heat thresholds (hotplate) and nociception scores (canonical discriminant analysis for hotplate, Hargreaves, dynamic von Frey) measured close to the time of tissue sampling were associated with the individual's ipsilateral brain AEA concentrations in TBI mice, but not in sham mice. The lower AEA, the lower was the threshold (Supplementary Figure S1C). Hence, although average nociceptive responses were inconclusive, low brain AEA levels appear to shift the nociceptive balance after TBI towards hypersensitivity.

### Non-goal directed hyperactivity associated with low plasma anandamide

**Figure 4:**
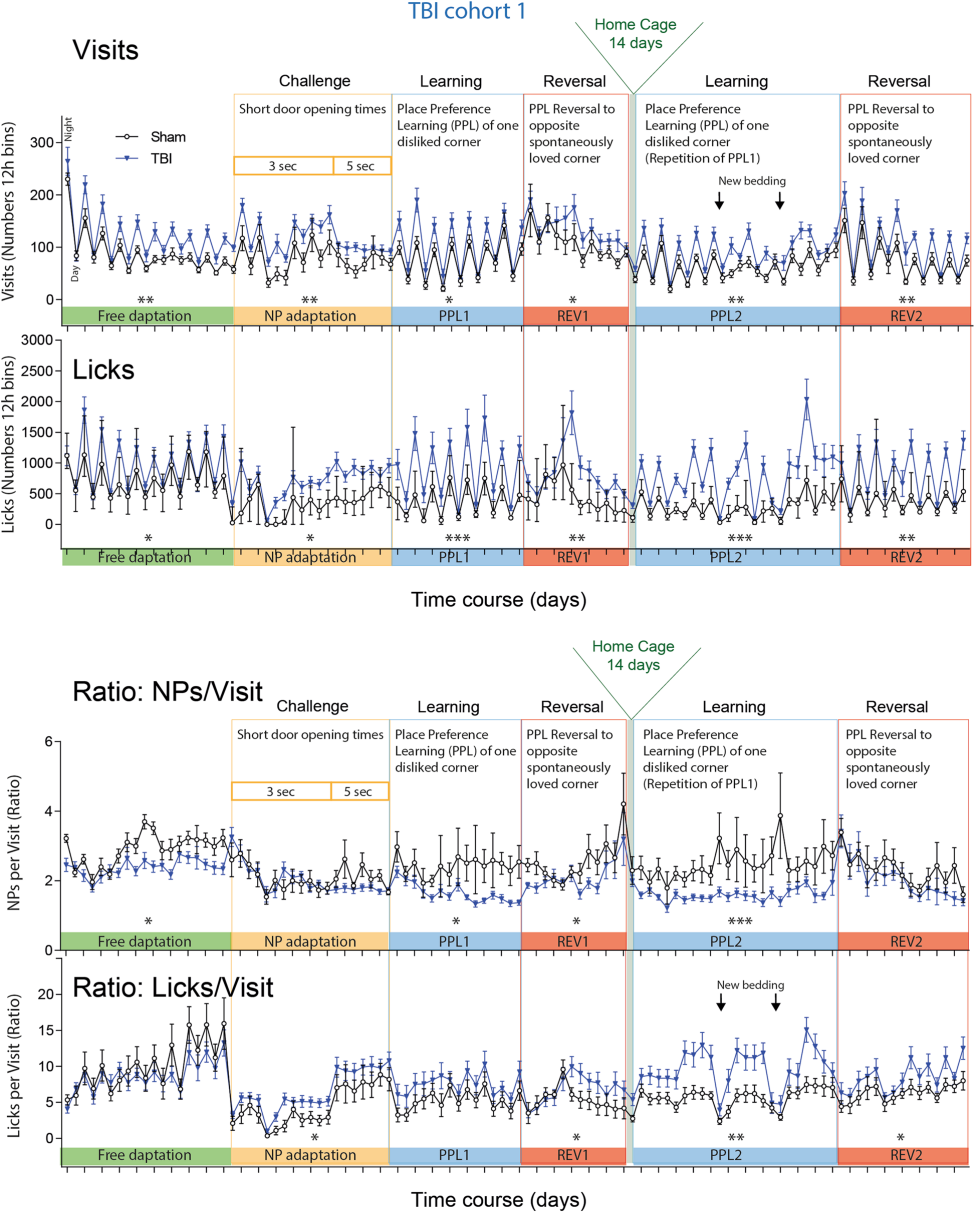
General activity of TBI versus sham mice in IntelliCages. Time courses of corner Visits and Lickings and ratios of Nosepokes (NP)/Visits and Licks/Visits in 12 h bins in TBI/sham cohort-1. The data show the mean ± SEM of n = 10 sham and n = 21 TBI mice. The peaks represent the nighttime activity. The tasks are explained in detail in Table 1 and abbreviation of parameters in Table 2. Tasks in which the time courses differed significantly between groups (2-way ANOVA for factor "group") are indicated per asterisks above the X-axis. *P < 0.05, **P < 0.01, ***P < 0.001. Data were analyzed in IntelliCage Plus software 2019 (XBehavior; https://www.xbehavior.com), exported as tab-separated txt files, imported in Microsoft Excel 2016, and Graphs were created with Graphpad Prism 8.4 (https://www.graphpad.com) and exported as emf. Graphs were arranged and labeled in Adobe Illustrator CC2020 (https://www.adobe.com/de), and exported to TIFF format.

The increase in response variability suggested fluctuations in behavioral performance, commonly observed post TBI^66^. We therefore studied longitudinal TBI evoked changes of activity, circadian rhythms, social behavior, learning and memory by using IntelliCages. The overview of the time courses of activity in 12 h intervals (Fig. 4) revealed a higher frequency of corner visits and licks throughout the observation of 54 days (cohort-1), which were more obvious during nighttime active periods (peaks in Fig. 4 upper panel), but circadian rhythms of daytime resting and nighttime activity were similar to controls. Disruptions of the circadian fluctuations were temporarily caused by short door opening times or learning tasks in both groups.

**Figure 5:**
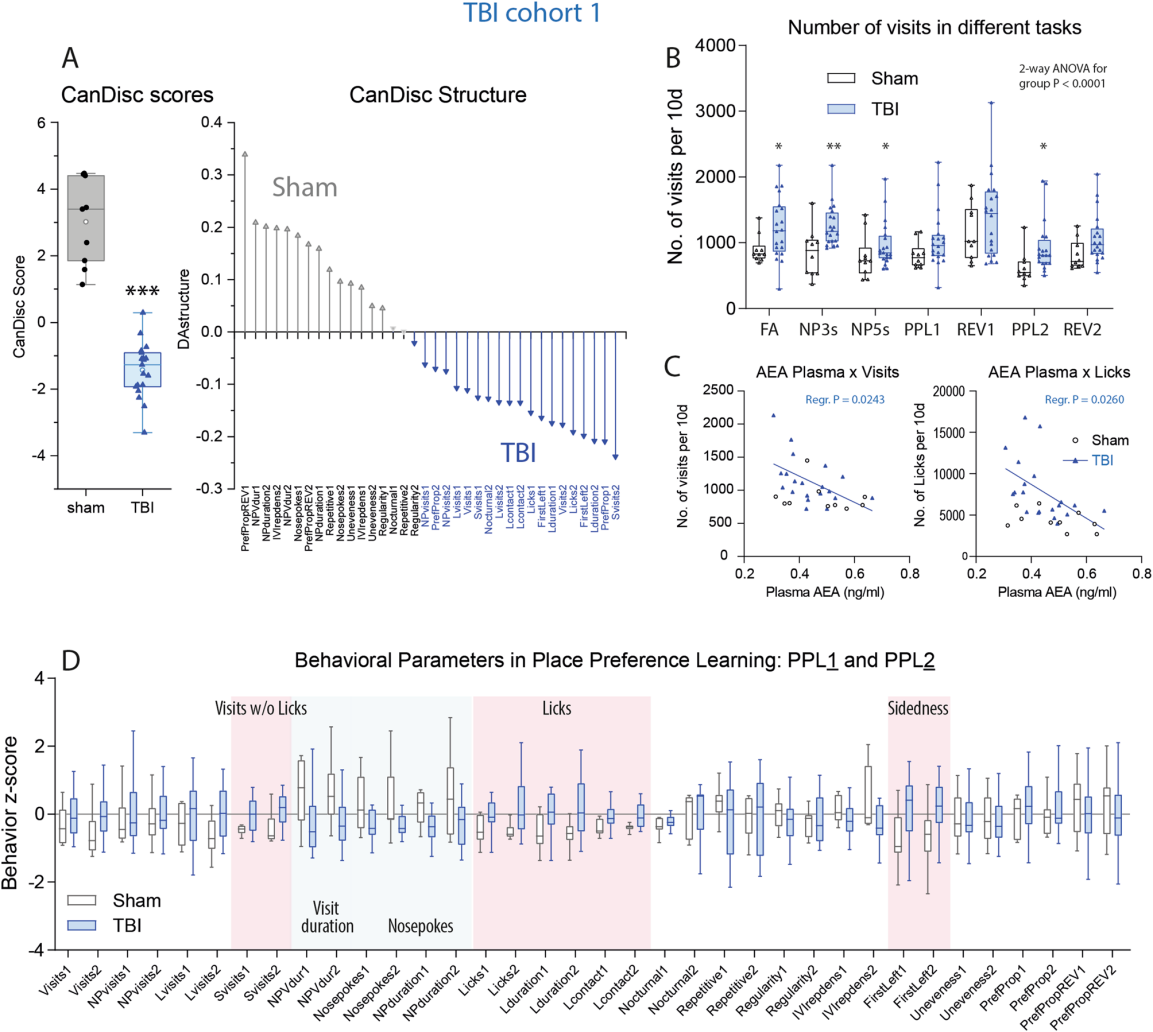
Canonical discriminant analyses of IntelliCage parameters, activity readouts in different tasks and behavioral z scores during place preference learning and reversal learning. (**A**) Canonical discriminant analysis score plot and structure. DA factor 1 explained 100% of the variance. The box shows the interquartile range, the line is the median, and the whiskers show minimum to maximum, each scatter is a mouse, the circle shows the mean. (**B**) Box/scatter plots of visits in different tasks normalized to periods of 10 days for each task. Each scatter is a mouse. n = 10 sham, n = 21 TBI; each two mice with incomplete data were excluded from the analysis. Data were compared with 2-way ANOVA for "group" × "task" and subsequent t test for group. *P < 0.05, **P < 0.01. Task abbreviations as in Fig. 4 and Table 1. (**C**) Linear association of the individual's average numbers of visits/10 day-periods and licks/10d versus the individual's plasma anandamide (AEA) concentration in the final blood sample. (**D**) Box plots of the z scores of behavioral parameters in PPL1/Rev1 and PPL2/Rev2. Parameters with "1" indicate PPL1/Rev1, "2" refers to PPL2/Rev2. Parameters, which were significantly increased in TBI mice, are highlighted in red, decreased parameters in blue (2-way ANOVA for "group", subsequent T-test for each parameter using an adjustment of P according to Šidák, P < 0.05, n = 10 sham, n = 21 TBI). Behavioral parameters and abbreviations are explained in Table 2. Behavioral data were analyzed in IntelliCage Plus software 2019 and FlowR 2017 (XBehavior; https://www.xbehavior.com), exported as tab-separated txt files, imported in Microsoft Excel 2016, and Graphs were created with Graphpad Prism 8.4 (https://www.graphpad.com) and exported as emf. Linear regression analysis (**C**) was done with Graphpad Prism. Graphs were arranged and labeled in Adobe Illustrator CC2020 (https://www.adobe.com/de), and exported to TIFF format.

Canonical discriminant analysis (CanDisc) was used to identify the behavioral parameters, which separated groups and predicted group membership (Fig. 5A). The CanDisc structure shows that visits, particularly those without licks (SVisits), licking parameters (numbers, contact time and duration), and left-sidedness were different between groups (Fig. 5A). The sidedness refers to the relative frequency of first left versus first right nosepoke (NP) upon corner visit, and showed that TBI mice turned preferably to the door on the non-injured side.

Summed visits over periods of 10 days showed the hyper-visiting activity after TBI in different tasks (Fig. 5B). The activity (visits and licks) was inversely associated with plasma anandamide levels (Fig. 5C) and with plasma OEA (not shown), i.e. the lower AEA, the higher was the activity. However, hyperactivity was not significantly associated with low body weights (Supplementary Figure S1D).

Behavioral z scores (Fig. 5D) were significantly increased for SVisits, all licking parameters and sidedness (red shaded areas in Fig. 5D), whereas the duration of visits with NPs (NPVdur) and number of nosepokes were reduced (blue shaded in Fig. 5D). The high frequency of non-goal-directed visits (SVisits) i.e. running in and out without NP and without licks suggests loss of attention, repetitiveness and agitation.

### Nighttime hyperactivity but normal daytime resting behavior

**Figure 6:**
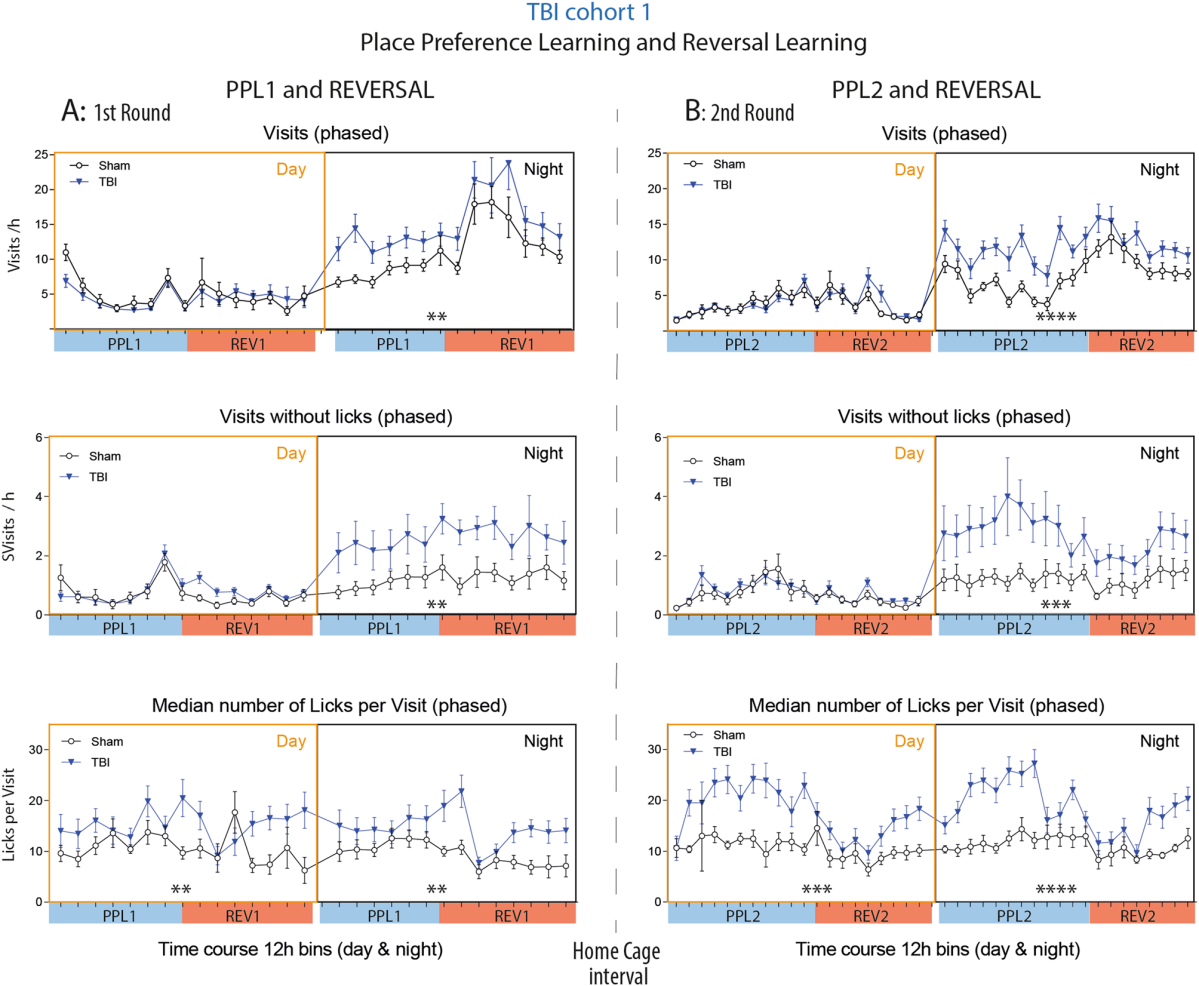
Phased activity of TBI versus sham mice in IntelliCages. (**A**,**B**) Time courses of corner Visits/h, Visits without Licks/h (SVisits) and Lickings per Visit in TBI/sham cohort-1 during the first (**A**) and second round (**B**) of place preference learning (PPL1, PPL2) and the respective reversal learning tasks (REV1, REV2), respectively. The data show the mean ± SEM of n = 10 sham and n = 21 TBI mice. One and two animals respectively dropped out during PPL1 or REV1 because of general health problems. SVisits are visits without attempt (nosepoke) to get reward. Asterisks above the X-axis indicate significant differences of the areas under the curve between groups as assessed by 2-way ANOVAs with the factors "group" and "time" (day or night), *P < 0.05, **P < 0.01, ***P < 0.001. Data were analyzed in IntelliCage Plus software 2019 and FlowR 2017 (XBehavior; https://www.xbehavior.com), exported as tab-separated txt files, imported in Microsoft Excel 2016, and Graphs were created with Graphpad Prism 8.4 (https://www.graphpad.com) and exported as emf. Graphs were arranged and labeled in Adobe Illustrator CC2020 (https://www.adobe.com/de), and exported to TIFF format.

The activity time courses (Fig. 4) suggested that daytime resting was less affected than nighttime active periods, and indeed, phased analysis of daytime and nighttime activity during the place preference learning and reversal learning tasks show that TBI mice were hyperactive in terms of visits during the night but were resting normally during the day (Fig. 6A,B). It is of note that the endocannabinoid system underlies a photoperiodic diurnal regulation^67^. AEA, OEA and PEA at highest in the dark phase whereas 1,2-AG raises in the light phase^68,69^ and is supposed to induce sleep^70^. The deficit of AEA, OEA and PEA may therefore manifest mainly in hyperactivity during the dark phase.

However, the median number of licks per visit on randomly successful visits (LVisits) was strongly increased at day and night. This overly licking, more than needed, is reminiscent of repetitive or compulsive behavior, or loss of feeling of satiation or feeding reward, which is contributed by dopamine—endocannabinoid circuits^59^.

### Night-time hyperactivity associated with low ipsilateral brain endocannabinoids

**Figure 7:**
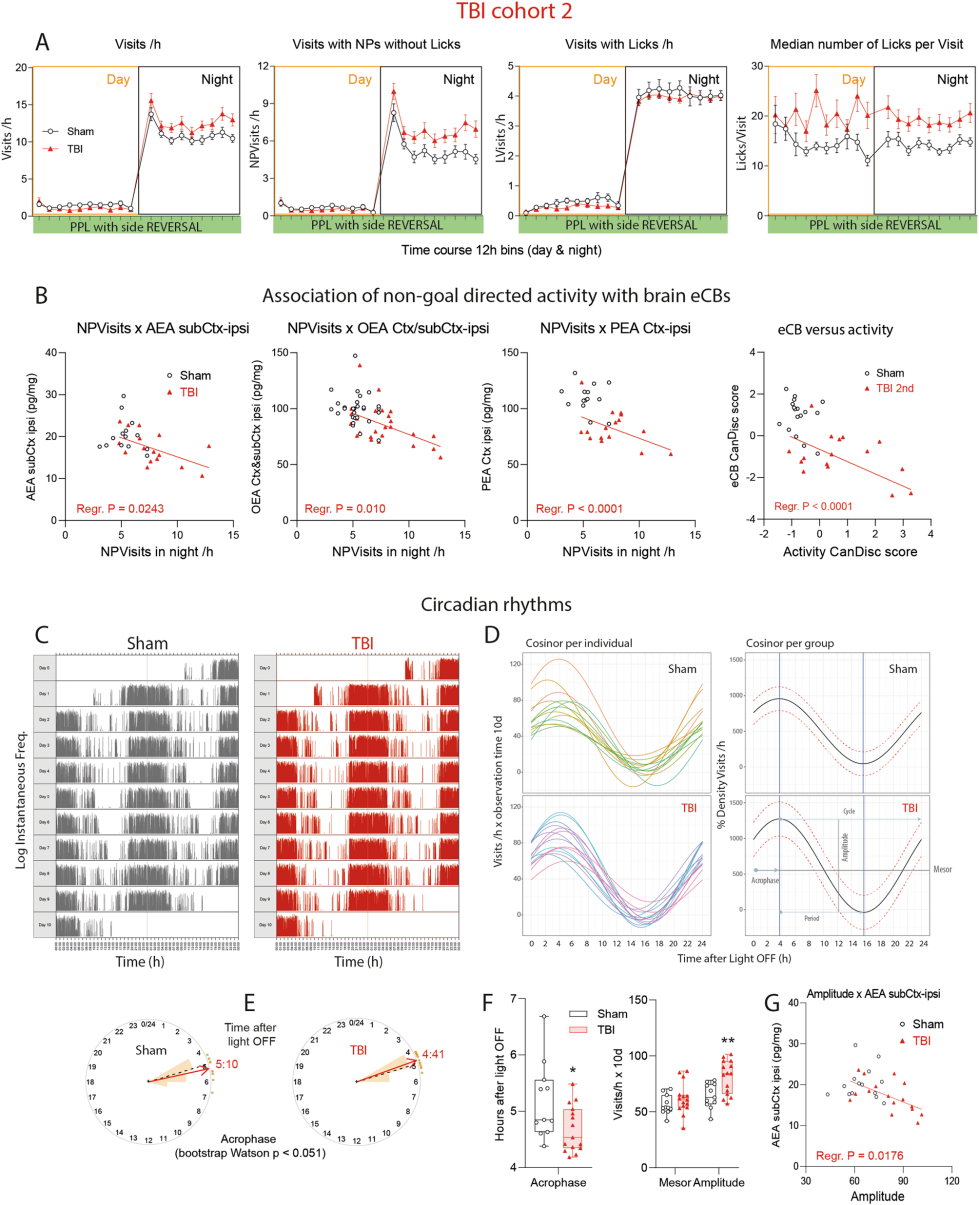
Phased behavior and circadian rhythms of TBI versus sham mice in TBI/sham cohort-2. (**A**) In analogy to Fig. 5 time courses of corner Visits/h, Visits with NPs without Licks/h (NPVisits), Visits with Licks (LVisits) and Lickings per Visit in TBI/sham cohort-2 during place preference learning (PPL) and reversal learning tasks (REV), with LED-based based decision making of the right side in the correct corner. Samples sizes were n = 15 per group. (**B**) Linear associations of the nighttime non-goal directed activity (NPVisits/h = visits with nosepokes but without licks per hour) versus endocannabinoid concentrations in the ipsilateral brain in cohort-2 and linear association of CanDisc scores for eCBs versus CanDisc scores of activity. Each dot is a mouse. For TBI mice, the slopes of the linear regression lines differed significantly from "zero" (no association for sham mice, P values given in the figure), i.e. hyperactivity was associated with low eCB. *Ctx* cortex perilesional, *subCtx* subcortical. (**C**) Exemplary actogram showing the circadian rhythms of corner visiting activity in TBI/sham mice of cohort-2 during place preference learning. The Y-axes show the logarithms of the instantaneous frequency, which is the reciprocal of the time from start of one visit to start of next visit. Analogous circadian data of cohort-1 in Supplementary Figure S2. (**D**) Cosinor analysis of visiting activity of individual mice and the groups. The circadian parameters are shown in the bottom right graph. The red dotted lines show the 95% CI. (**E**) Circular presentation of the acrophases i.e. the time from Light OFF to maximum activity. (**F**) Quantitative and statistical comparison of major circadian parameters, acrophase, mesor and amplitude, as graphically depicted in (**C**) Box plots shows the interquartile range, the line is the median, the whiskers show minimum to maximum, dots are individual mice. Asterisks indicate significant differences between groups (2-sided, unpaired t tests; *P < 0.05, **P < 0.01, n = 15). (**G**) Linear association of the circadian amplitude versus AEA concentration in the ipsilateral brain underneath the lesion (subCtx ipsi). For TBI mice, the slope of the line differed significantly from "zero" (no association for sham mice). High amplitude was associated with low AEA. Data were analyzed with IntelliCage Plus software 2019 and FlowR 2017 (XBehavior; https://www.xbehavior.com), and images in (**C**–**E**) were exported from FlowR as .png, and actograms in (**C**) were colored to fit the groups (grey and red) in Adobe Photoshop CC2020. exported as tab-separated txt files, imported in Microsoft Excel 2016, and Graphs were created with Graphpad Prism 8.4 (https://www.graphpad.com) and exported as emf. Linear regression analyses (**B**,**G**) were done in Graphpad Prism. Graphs were arranged and labeled in Adobe Illustrator CC2020 (https://www.adobe.com/de), and exported to TIFF format.

We confirmed the hyperactivity and over-licking in the second cohort of TBI and sham mice (Fig. 7A). Indices of non-goal directed hyperactivity i.e. visits without licks were inversely associated with the individual's endocannabinoid levels in the ipsilateral brain (Fig. 7B). The lower AEA, OEA and PEA, the stronger was the hyperactivity. There was no such association for sham mice, which clustered in more homogenous clouds (3D-plots in Supplementary Figure S1E). The hyperactivity was also evident in analyses of circadian rhythms (Fig. 7C–G for TBI/sham cohort-2; Supplementary Figure S2 shows cohort-1). Actograms (Fig. 7C), cosinor analysis (Fig. 7D) and bootstrap analysis (Fig. 7E) clearly revealed the higher amplitude in TBI mice, with about 30 min earlier time of maximum activity (acrophase). Statistical results are in Fig. 7F. Comparison of cohort-2 (Fig. 7) with cohort 1 (Supplementary Figure S2) revealed astonishingly similar acrophases in TBI mice, about 4.5 h after light OFF. The amplitudes were significantly higher in both TBI cohorts, versus the respective sham animals, and the amplitudes were inversely associated with ipsilateral brain AEA levels, i.e. the lower AEA, the higher was the amplitude (Fig. 7G).

### No deficit in reward based learning but faster extinction of avoidance memory

**Figure 8:**
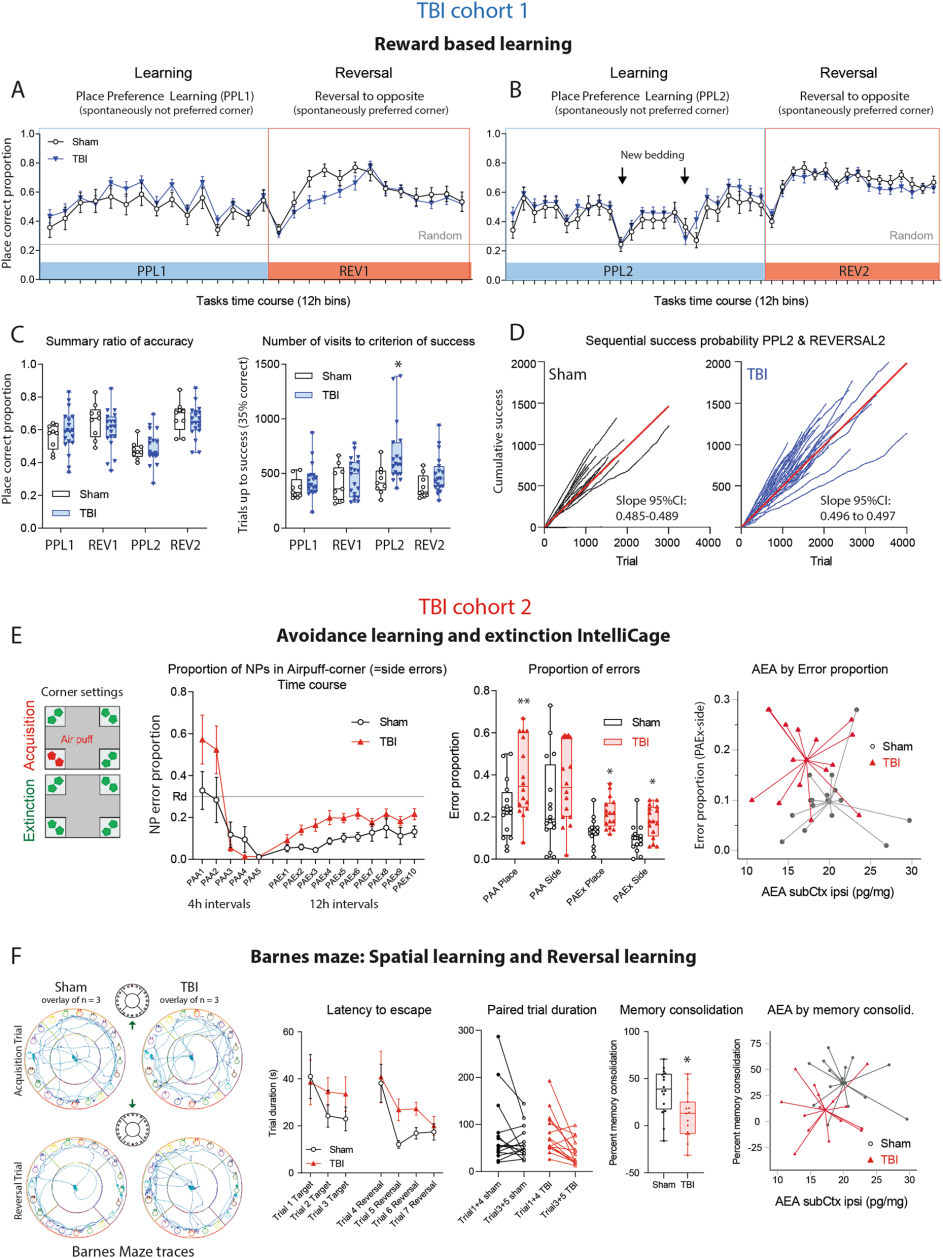
Learning and memory of TBI versus sham mice in reward- and avoidance-based tasks. (**A,B**) Time courses of the proportion of correct corner visits during place preference learning and reversal learning tasks in TBI/sham cohort-1. During the learning period (PPL1, PPL2) mice had to prefer a spontaneously infrequently used corner (more difficult), the Reversal (REV1, REV2) switched the rewarding corner to the opposite side to a spontaneously preferred corner. The expected random correctness is 0.25. The time courses did not differ between groups (2-way ANOVA). The samples sizes were n = 10 sham, n = 21 TBI. One and two animals respectively dropped out during PPL1 or REV1 because of general health problems. (**C**) Box/scatter plots showing the individual's mean proportion of correctness and number of trials needed to reach the criterion of success during the place preference learning (PPL1, PLL2) and reversal learning (REV1, REV2) tasks of TBI/sham mice of cohort-1. Sample sizes as in (**A**,**B**). The box shows the interquartile range, the line is the median, the whiskers show minimum to maximum, dots are individual mice. During PPL2, olfactory cues were removed twice by changing the bedding material (time points indicated in **B**). Correctness dropped similarly in both groups, but TBI mice needed more trials to success during this period. The criterion of success was 35% correctness, i.e. 10% above random. Data were compared with 2-way ANOVA with the factors "group" and "task" and subsequent posthoc t test for each task using an adjustment of alpha according to Šidák, *P < 0.05. (**D**) Sequential success probability plots exemplarily shown for PPL2/REV2. The cumulative correct responses plotted versus trial number for each animal, and the steepness of the linear regression line (red) shows the learning velocity. The 95% confidence intervals did not overlap, suggesting faster learning of sham mice during this period in agreement with 2C. The plot reveals the hyperactivity of TBI mice. (**E**) Place avoidance acquisition (PAA) and extinction (PAEx) in TBI/sham cohort-2 using an airpuff punishment in one corner on both sides during acquisition, followed by a 24 h home cage interval, and no reinforcement during extinction except LED reminder (illustration left). Time courses show the means and SEM of n = 15 mice per group. The box/scatter plots show the individuals' mean proportion of place and side errors during the PAA and PAEx periods. "Place error" is a visit to the forbidden corner, and "Side error" is a nosepoke in the forbidden corner. Data were compared with 2-way ANOVA with the factors "group" and "task" and subsequent posthoc t test for each task using an adjustment of alpha according to Šidák, *P < 0.05, **P < 0.01. The right XY-scatter plot with centroid spikes shows the group separation based on AEA in subcortical ipsilateral brain tissue (AEA subCtx ipsi) versus side errors during extinction. There was no association of AEA by errors. (**F**) Barnes maze path tracks and learning behavior in TBI/sham cohort-2, late after surgery (5–6 months, n = 16 sham, n = 15 TBI). The left shows the superposition of path tracks of each three mice of the first learning trial and first reversal trial. The time courses of the latency to escape were similar at baseline and dropped in both groups, but dropped faster in sham mice than TBI mice. Hence, memory consolidation defined as percentage change of the escape latency of learnt trials (Trial 3, 5, 6) versus baseline trials (Trial 1, 4) was lower in TBI mice (2-sided unpaired t test, *P < 0.05). The right XY-scatter plot with centroid spikes shows the group separation based on AEA in subcortical ipsilateral brain tissue (AEA subCtx ipsi) versus memory consolidation. There was no association of AEA by memory consolidation. Data in (**A**–**E**) were analyzed with IntelliCage Plus software 2019 and FlowR 2017 (XBehavior; https://www.xbehavior.com/), exported as tab-separated txt files, imported in Microsoft Excel 2016, and Graphs were created with Graphpad Prism 8.4 (https://www.graphpad.com) and exported as emf. Barnes Maze behavior was analyzed with EthoVision XT (https://www.noldus.com) and exported to Excel. Spiked scatter plots in (**E**,**F**) were created in SPSS 25 (https://www.ibm.com/de) and exported as emf. Graphs were arranged and labeled in Adobe Illustrator CC2020 (https://www.adobe.com/de), and exported to TIFF format.

FFAH inhibition has been reported to improve the performance of head-injured mice in classical maze tasks of spatial memory^27^, suggesting that low brain eCBs would impede learning. We assessed learning and memory in reward-based and avoidance-based tasks in the IntelliCage and Barnes Maze. In IntelliCage place preference learning (PPL1, PPL2) and reversal learning (PPLrev1, PPLrev2), TBI mice were not inferior to sham mice (Fig. 8A–D). All mice reached about 60–70% of accuracy (Fig. 8C) and needed similar numbers of trials to reach the criterion of success, which was set to 35% accuracy i.e. 10% above random. The first set (PPL1/PPLrev1) and second set (PPL2/PPLrev2) were done sequentially with a home cage interval of 14 days. In PPL2, mice were "experienced" but started without olfactory cues, and in this PPL2, TBI mice needed more trials to success, suggesting reduced long-term memory. However, the time courses did not differ significantly. The cumulative learning curves again reveal the hyperactivity of TBI mice but the steepness (i.e. velocity of learning) was similar (Fig. 8D).

In avoidance learning, mice had to learn to avoid one corner, where NP was punished with an airpuff, and water access was denied (PAA). During extinction (PAEx), mice had to remember not to use this corner. There was no reinforcement, but LED was a reminder (Fig. 8E). Place avoidance acquisition (PAA) is an easy task and all mice learnt not to make NPs in the forbidden corner. The proportion of side errors dropped to < 10% at the end of the acquisition period, but TBI mice visited the forbidden corner more frequently (place errors). During extinction, TBI mice made more place errors (wrong visits) and side errors (wrong NPs) than the controls showing a faster loss of avoidance memory and lower attention to LEDs. Although XY-scatter clouds of AEA versus error proportions clearly separated TBI mice from sham mice, there was no linear or non-linear relation between eCBs and errors (Fig. 8E right).

Spatial learning in the Barnes maze (Fig. 8F) relies on the natural shelter seeking behavior. Baseline latencies and paths to escape were similar in both groups. Upon daily repetitions, sham mice got faster with each trial. This memory consolidation also occurred in TBI mice but required more trials. The overall performance of TBI mice in Barnes maze was not compromised. Again, memory readouts versus endocannabinoids clearly separated the groups but without significant relation between AEA (or OEA, PEA) with memory consolidation (Fig. 8F right). Hence, in contrast to the hyperactivity, cognitive performance in these tasks was not directly related to brain eCBs.

## Discussion

We show in the present study that endocannabinoids of the fatty acid ethanolamide group (AEA, OEA and PEA) are persistently reduced in the ipsilateral perilesional and subcortical brain after TBI, and that low levels are associated with persistent non-goal directed nighttime hyperactivity and over-licking. The lower the eCB, the stronger was the hyperactivity.

In light of previous studies showing that CB1 agonists improve survival and outcome in TBI patients and reduce neuroinflammation and neuronal excitotoxicity in rodent TBI models^25,71–73^, the observed lasting loss of endocannabinoids discloses an underlying mechanism, and supports the idea that inhibition of FAAH, the enzyme catalyzing the breakdown of AEA, OEA and PEA, could be a novel therapeutic approach. In rodent models, FAAH inhibitors attenuated TBI-evoked inflammatory markers in association with better performance in balance beam walking and Morris water maze^27,28^. The observed deficits of eCBs in the post-TBI brain give a biological substantiation for the use of pro-cannabinoid treatments after TBI, which may not only reduce the pathology in the early phase after TBI^31^ but also long-term mal-adaptations that manifest in psychopathological behavior. Attention deficit disorder and PTSD are highly prevalent after TBI^4^ and responsive to pro-cannabinoid medication^33,74,75^.

The strongest behavioral abnormality of TBI mice was non-goal directed hyperactivity, i.e. an increase of visits without licks or nosepokes, without thorough exploration and without attempt to get something to drink. This non-expedient "running-in-and-out" behavior also occurs in models of dementia^76^ and is reminiscent of behavior of attention deficit hyperactivity disorder (ADHD)^77^, which has been described as one of the many post-TBI disabilities in humans^78,79^. The association of human ADHD with traumatic brain injury appears to be bidirectional, i.e. a pre-injury trait and a post-injury disorder^79^. In our experiments, mice were from one vendor, were randomly assigned to sham or TBI, and evenly allocated to the IntelliCages. Hence, we infer that the hyperactivity and attention deficit behavior is a consequence of the brain injury and interpret the behavior as a correlate of the post-traumatic stress and disease and difficulty to make economic value-based choices.

The linear relationship of low brain eCBs with nighttime hyperactivity, also manifesting in high circadian amplitudes, is suggestive of a causative nature. The results would agree with the photoperiodic diurnal predominance of AEA, OEA and PEA in the dark phase^67^ so that deficits have stronger impact on nighttime activity. A limitation is that the tissue was sampled in light. There was no such association of eCBs with other behavioral parameters, although these parameters also clearly separated TBI from sham mice, including body weights, avoidance memory and sidedness. AEA and OEA regulate appetite and satiety^50,60^, likely inversely, but neither brain, nor plasma levels were associated with body weights, and body weights not significantly with activity, suggesting that lower body weights were not a consequence of eCB deficiency (alone) or of high physical activity and vice versa.

In spite of the large cortical defect and persistently low brain endocannabinoids, TBI mice had no overt deficits in locomotion and motor coordination, but a persistent left-sidedness meaning that they preferentially turned to the left door upon corner entry in the IntelliCage, away from the cortical injury side. Sidedness was not associated with right or left-brain endocannabinoids or the difference between both sides (not shown), but was a robust feature in both TBI-cohorts and may point to a form of agnosia. It is conceivable that sidedness has masked right versus left differences of somatosensory responses. Therefore, hotplate responses, which do not differentiate between right and left side, were most reliable, and these responses were linearly associated with ipsilateral brain AEA levels in TBI mice (not in sham). The lower AEA, the lower was the withdrawal latency suggesting a weakening of inhibitory "pain" control, considering the individual response.

Average heat-stimulated paw withdrawals were however within normal limits. There was a substantial increase of the inter-individual and intra-individual variability suggesting a plethora of somatosensory alterations from sensory loss to hypersensitivity and fluctuations of attention, which have been described after TBI^66^. As a group, TBI mice did not show nociceptive behavior suggestive of central pain or headache, neither early nor late after the injury, which agrees with previous studies, in which TBI had to be combined with additional nociceptive stimuli to reveal a hypersensitivity^63,80^, except in a study where TBI mice showed stronger heat-evoked avoidance behavior in an operant orofacial test 2 weeks after TBI^64^. The test relies on conflict behavior i.e. decision if the reward was worth tolerating the nociceptive stimulus, hence rather suggesting alterations of value-based decision-making. The observed repetitive over-licking in our study may also point to changes of reward salience, which depends on dopamine and endocannabinoid reward circuits^59^, and elicits descending pain inhibitory signals^16^.

Indeed, low AEA was associated with particularly high numbers of licks during successful visits (LVisits) with long licking duration and long bottle contact time. This over-licking was evident day and night and maintained over months. Overly licking suggests compulsiveness or loss of appreciation of reward that depends on endocannabinoids^81^. Alternatively, the behavior may be indicative of a stronger need owing to hyperthermia—unlikely to resist over months—or higher water loss. The drinking water was just noticeably sweet to increase the learning drive, which does not elicit compulsive behavior in C57Bl6 mice. In TBI patients, renal sodium and water loss and cerebral salt wasting are TBI complications^82,83^, but the extent of over-licking in our mice was far from being a health problem such as sodium wasting. So overall, in spite of the translational limitations, the behavior suggests a reduced appreciation of satisfaction. It is important to note, that the major behavioral phenotypes, in particular over-licking and hyperactivity, were observed and confirmed in two independent sequential TBI cohorts, and in both cohorts it was associated with low AEA in plasma or ipsilateral brain.

In agreement with previous studies in mice carrying a cortex-lesion^84^, TBI mice were not inferior to sham mice in reward-based learning and reversal learning tasks, but showed lower maintenance or consolidation of memory in avoidance-based tasks. It has to be considered that the IntelliCage provides an enriched environment, which is known to improve the recovery after TBI^85^ suggesting that we look at behavior under these enrichment-provided therapeutic conditions. Although XY-scatter plots of "avoidance errors" versus AEA (or PEA) separated TBI mice clearly from sham mice, there was no direct association of errors with low eCB. It has been shown that FAAH-inhibition after TBI in mice reduces the escape latency in a Morris water maze, interpreted as CB1 mediated improvement of aversive spatial memory^27^. On the other hand, endocannabinoids contribute to the active suppression of threatening memories in humans^86^ and rodent PTSD models^39,87,88^, suggesting that dual effects in avoidance-based tasks might have masked the impact of the endocannabinoid system in our tests. The predictable and avoidable airpuff in one corner does not elicit fear, and faster re-usage of this corner during extinction suggests faster loss of memory rather than faster loss of anxiety.

Although the protracted behavioral consequences particularly in terms of motor function and cognition were astonishingly mild in TBI mice and much different from serious consequences of TBI in humans, the behavioral phenotype points to posttraumatic hyperactivity with attention deficit in clear association with low brain endocannabinoid levels suggesting that pro-cannabinoid directed therapeutic or preventive approaches may improve post-TBI psychopathology and encephalopathy. In particular, the persistent loss of endocannabinoids in cortex and sub-cortex substantiates approaches, which enhance the endogenous endocannabinoid levels.

## Methods

### Mice

Female C57BL/6N mice were subjected to a traumatic brain injury (TBI) at the age of 7–9 weeks using the controlled cortical impact (CCI) method^89^ or sham surgery. Female mice were used because behavioral studies in IntelliCage rely on observations of social groups of 16 mice who live together in one large cage. Sham controls underwent sham surgery consisting in anesthesia, stereotactic mounting, skin incision and closure. The skull remained intact. All mice were housed and maintained in a controlled environment (12 h dark/light cycle, 22 ± 2 °C, 45–60% humidity) with food and water ad libitum. Mice were randomized and experimental performance and data acquisition were done in a blinded and unbiased fashion. The experiments followed the "Principles of laboratory animal care" (NIH publication No. 86-23, revised 1985). They were approved by the local Ethics Committee for animal research (Darmstadt, Germany; FK-1094) and the Animal Care and Ethics Committee of the Landesuntersuchungsamt Rheinland-Pfalz (#G12-1-052), adhered to the guidelines for pain research in conscious animals of the International Association for the Study of PAIN (IASP) and were in line with the European and German regulations for animal research and the ARRIVE guidelines.

### Experimental traumatic brain injury by controlled cortical impact (CCI)

Experimental TBI was performed using the controlled cortical impact (CCI) model essentially as described^90^. Briefly, anesthesia was induced with 4% (v/v) isoflurane and maintained with 2% (v/v) isoflurane, mice were fixed in a stereotactic frame and a craniotomy (4 × 4 mm) was drilled with a handheld dentist's drill. The brain was exposed to an electromagnetic driven impactor (Leica) and TBI was induced to the right parietal cortex using the following parameters: diameter impactor tip 3 mm, velocity 6 m/s, duration 200 ms, depth of penetration 1.5 mm. Sham operated mice served as controls. For maintaining physiological temperature of 37 °C, the animals were kept on a feedback‐controlled heating pad (Hugo Sachs) and for optimal recovery transferred to an incubator (Draeger) for 1.5 h after surgery. The study was done with two cohorts of TBI mice and the respective two cohorts of sham mice. The first part encompassed 20 TBI and 10 sham mice, the second each 16 mice. The experiments were done sequentially. Four TBI and two sham mice dropped out during experiments because of general health deterioration.

### Assessment of blue light sensitivity in posttraumatic headache

Blue light sensitivity was assessed as described^61^. A plexiglass test chamber (25 × 10 × 15 cm) was divided into 3 zones, i.e. red, blue, and neutral in the middle. Mice were allowed to move freely in the chamber. By entering the unlit red or the blue zone, the red (625 nm) or blue (460 nm) illumination (0.35 mW/cm^2^ light intensity) was switched on. Illumination was maintained until the mouse left the respective zone or after 15 s if the mouse did not leave. Presence in the neutral zone did not trigger illumination. Recording started three seconds after placing the mouse into the neutral zone and stopped after completing the trial of 10 min. Behavioral data were recorded automatically including the time in the zones, number of visits of the zones, distance moved and the number of switches using EthoVision XT (Noldus).

### Analysis of nociception and motor function

All behavioral tests were performed by one investigator in a blinded and randomized fashion as described previously^76,91–94^. Mice were habituated to the test room and test cages for three consecutive days. The latency of paw withdrawal on mechanical stimulation was assessed using a dynamic von Frey apparatus (Aesthesiometer, Ugo Basile) employing a force range of 0–5 g, ramp of 0.5 g/s and hold at 5 g until paw withdrawal. The sensitivity to painful heat stimuli was assessed by recording the paw withdrawal latency in the Hargreaves test (IITC Life Science), which employs a radiant heat source targeted to the plantar surface of the hind paw with help of a mirror system. The lamp emits a heat beam until the paw is withdrawn. Additionally, the hotplate test was performed where mice were placed on a surface heated to 52 °C and the latency to behavioral indication of nociception i.e. lifting, licking or jumping was measured. To assess the impact of TBI on motor function, the Rotarod test was performed, where mice were placed on a rod spinning at 36 rounds per minute and the latency to fall was measured. The cut-off time was set at 180 s on a constant speed Rotarod, and 300 s on an accelerating Rotarod.

### Learning and memory

#### IntelliCage setup

The IntelliCage (NewBehavior AG, Zurich, Switzerland, TSE systems)^95^ consists of four operant corners, each with two water bottles, sensors, light emitting diodes (LEDs) and doors that control the access to the water bottles. The system fits into a large cage (20 × 55 × 38 cm, Tecniplast, 2000P). Four triangular red shelters (Tecniplast) are placed in the middle to serve as sleeping quarters and as platforms to reach the food. The floor is covered with standard bedding.

Mice are tagged with radio-frequency identification (RFID)-transponders, which are read with an RFID antenna integrated at corner entrance. Inside the corners, there are two holes with water bottles, which can be opened and closed by automated doors. Mice have to make nosepokes (NP) to open the doors for water access (tasks in Table 1). The IntelliCage is controlled by the IntelliCage Plus software, which executes pre-programmed experimental tasks and schedules. The numbers and duration of corner visits, nosepokes, and licks are automatically recorded without the need for handling of the mice during the recording times (summary of behavioral parameters in Table 2). Sixteen mice were housed per cage.

**Table 1:** Task description of IntelliCage experiments.

Task	Protocol	Aim	Punish	Duration of the protocol in days (d)	
				Cohort-1	Cohort-2
Free Adaptation	FA	Habituation, no tasks, all doors open	No	4d	14d
Nosepoke adaptation	NP	NP opens doors for 5 s. Learn to make NPs to open doors	No	10d	45d
Nosepoke 50% probability	NP2s-door opening probability 50%	NP opens door for 2 s, with a probability of 50%	No		7d
Place preference learning	PPL	Water/sugar water access on NP in one corner, learn to prefer the awarded corner, which was a spontaneously not-preferred corner, 7 or 8 mice assigned to one corner to avoid competition	No	7d, 12d	
Place preference reversal	PPLrev	Switch of rewarded corner to opposite side, relearn corner preference, the switch was to a spontaneously preferred corner	No	6d, 7d	
Place avoidance acquisition	PAA	NP in one corner punished with air-puff, and doors remain closed in this corner, associated with red LED. Learn to avoid this corner	Air-puff		1d
Place avoidance extinction	PAEx	NP protocol, no restrictions, no air-puff, but corner assignment as during acquisition. Assess extinction of aversive memory	No		5d
Place preference Learning with side decision	PPL-SD	Water in only one corner on one side, randomizer determines “correct side”, correct NP opens door for 5 s, correct/incorrect sides switch after 50 correct visits, correct side is lit with green LED	No		7d
Place preference Reversal Learning with side Reversal	PPLrev-SDrev	Switch to opposite corner, incorrect side lit with red LED	No		7d

**Table 2:** Parameters and abbreviations of IntelliCage behavior.

Visits	Visits/h
NPvisits	Visits with Nosepoke without licks/h
Lvisits	Visits with Licks/h
SVisits	Visits without Licks and without Nosepokes/h
NPVdur	Median duration of Visits with NP w/out Lick (s)
Nosepokes	Mean number of Nosepokes during Visits with NP w/out Licks
NPduration	Median duration of such Nosepokes during a Visit (s)
Licks	Median number of Licks per Visit
Lduration	Median duration of Licking during a Visit (s)
Lcontact	Median bottle cap contact time during a Visit (s)
Nocturnal	Log(Visit frequency during dark phase/Visit frequency during light phase)
Repetitive	Repetitiveness, log(sum of observed returns to same corner/sum of expected such switches)
Regularity	Sqrt (sum of sq non-diag. transition matrix residuals/sum of non-diag. transition matrix observed values)
IVI	Intervisit intervals (s) i.e. time from end of visit to start of next corner visit
IVIrepdens	Intervisit intervals (s) for repeated use of the same corner
InstantFreq	Instantaneous frequency, reciprocal of the time from start of one visit to start of next visit
Unevenness	Describes the relative use of corners, ranges from 0 to 1 (0 = equal use of 4 corners, 1 = exclusive use of 1 corner)
Sidedness	Ratio of visits with first left versus first right NP of visits with NPs
Mesor	Midline estimating statistic of rhythm, a rhythm adjusted mean in cosinor analysis
Amplitude	Difference between Mesor and Peak activity
Acrophase	Time to maximum activity after Light Off (Light off set to 0)
Period	Duration of one cycle

#### IntelliCage learning tasks

The time schedule for the IntelliCage experiments is shown in Fig. 4, and the experiments followed protocols described in our previous studies^61,76,96,97^ and were adapted to TBI mice. They are summarized in Table 1. TBI and sham mice were pseudo-randomly assigned to the cages to have equal numbers per group per cage. They were adapted to the system with free access to every corner, with all doors open, water and food ad libitum. The free adaptation (FA) was followed by "nosepoke adaptation" (NP), during which the doors were closed, the first nosepoke of the visit opened the door for 5 s. To drink more, the animals had to leave the corner and start a new visit. The NP protocol was maintained in subsequent tasks. An NP 2 s protocol with 50% door opening probability was intermittently used in the second part of the study to increase overall activity.

In the “place preference learning” (PPL) task mice had to learn to prefer one corner, where they were rewarded by water/sugar water access. Each seven or eight mice were assigned to one corner to avoid competition. After conditioning to the corner, PPL reversal learning (PPLrev) was assessed by switching the rewarding corner to the opposite side. First, mice were assigned to one corner, which they infrequently used spontaneously. The switch then allowed them to use a spontaneously preferred corner. The PPL and respective Reversal Learning tasks were repeated with the same settings but with a home cage interval of 14 days in between.

In the Place Avoidance Acquisition (PAA) task (as described e.g. in^61,97^, mice learnt to avoid one corner, where a nosepoke triggered a red LED and an air-puff (1 s; 0.8 bar) from above the head. The door remained closed so that water access was denied. In the three remaining corners, the mice were allowed to drink for 5 s upon NP. At the end of the 24 h acquisition period, mice were removed to their home cages for 24 h and deprived of water for the last 16 h before returning to the IntelliCage. In the following 5 days, extinction of the aversive memory was assessed (PAEx), in which all doors opened in response to a nosepoke and no air-puff was applied. Only the red LED still reminded of the previously punished corner.

In the subsequent PPL-Side Decision (PPL-SD) task, the animals learned to drink in one correct corner on one side, and to make the correct decision of the side according to green (correct) or red (incorrect) LED. The task was divided into two alternating modules with switches after 50 correct NPs. The mice started in the “SDcorrect” protocol where a green LED was switched on at the correct side, the other side was unlit. Once the task was successfully completed 50 times, the protocol switched to “SDincorrect” where a red LED marks the wrong side (the other side remains unlit). The right and wrong sides of one corner were randomly assigned for each visit.

### Barnes Maze spatial learning and memory

Female mice of cohort-2 were used for analysis of spatial learning (TBI n = 15; Sham controls n = 16) in a Barnes Maze (TSE, Bad Homburg, Germany) after completing experiments in the IntelliCage. They were 27 weeks old at start of Barnes Maze adaptation. A reward-based 2-choice-Barnes Maze paradigm and a classical avoidance-based Barnes Maze were employed sequentially. For both protocols, the maze was divided into five zones: center, target (i.e. rewarding box), opposite, positive and negative. The protocols consisted of three trial phases: habituation, target acquisition, and reversal learning. In the habituation phase, mice were placed in the middle of the maze under a transparent cylinder for 30 s, and were then guided to the target hole, where they were allowed to enter the shelter within 3 min. If they did not enter the escape box within this time, they were gently nudged into it and allowed to stay there for 1 min. For the 2-choice-Barnes Maze, two boxes were placed on opposite sides of the maze. One of the two boxes, the target box, contained sweet rewards for the mice, which were kept on a mild reduction diet (1.5 g pellets/mouse/day) to increase motivation. The animals were introduced to both boxes during habituation, and they were supposed to prefer the reward box. The other was empty. The habituation was performed on three consecutive days with two trials per day. In the Target Acquisition phase (4 days for 2-choice, 3 days for classical, one trial per day), mice were placed in the middle of the maze under an opaque cylinder for 30 s and then allowed to freely explore the maze for 5 min to find and enter the escape box. In the subsequent Reversal Learning phase (4 days, 1 trial per day) the escape box or the treat box were moved to the opposite side of the maze. EthoVision XT 11.5 software (Noldus) was used for video tracking and analysis. Overlays of path tracks were created in Adobe Photoshop CC2020. Trial duration, distance moved, velocity, zone visits and cumulative duration in each zone was recorded.

### Immunofluorescence and histology

Immunofluorescence analyses utilized final tissue of TBI/sham cohort-1, obtained 101 days after TBI. Mice were terminally anaesthetized with isoflurane and transcardially perfused with cold 0.9% saline followed by 4% paraformaldehyde (PFA) in 0.9% saline for fixation. Brains were excised and post fixated in 4% PFA. Brains were embedded in low-temperature melting agarose and cut using a vibratome (Thermo Scientific). Coronal sections (80 µm) were collected from bregma − 0.94 to bregma − 2.92 in PBS. For immunohistochemistry, sections were blocked in PBS, 5% normal goat serum, 0.5% bovine serum albumin, 0.3% Triton X-100 and incubated with the primary antibodies rabbit anti-Iba1 diluted in blocking solution (Wako Chemicals, 1:1,500) and rat anti-GFAP (Life Technologies, 1:1,000) overnight at 4 °C, followed by excessive washing in PBS/0.1% Triton X100 and incubation with secondary Alexa Fluor 488 or 568 conjugated secondary antibodies (Life Technologies, 1:1,000 dilution). Images of the brain sections were captured using a confocal microscope (LSM510, Zeiss) and equal acquisition parameters. Images were exported from LSM Image Browser 4.2. to Adobe Photoshop CC2018 for a global adjustment of color balance. Images were digitally processed using appropriate threshold settings for background reduction and the numbers of Iba1 and GFAP immunolabeled cells was determined using the ImageJ Particle Counter plugin (FIJI ImageJ^98^) after background subtraction and threshold setting using auto-detection with minor adjustments essentially as described in^89^.

### Analysis of endocannabinoids

Endocannabinoids were analyzed in plasma in TBI/sham cohort-1 using final blood samples 101 days after surgery, and in brain tissue at the end of the observation period in cohort-2 (155 days after TBI). Lipid analyses were done using liquid chromatography–electrospray ionization–tandem mass spectrometry (LC–ESI–MS/MS), according to procedures we have developed and described in e.g.^91,99,100^, recent updates in^101,102^.

Plasma sample (100 μl) or tissue homogenates were extracted by liquid–liquid-extraction using two cycles of ethyl acetate: n-hexane (9:1, v/v) after spiking them with the corresponding internal standards (deuterated analytes). The analysis was performed using a hybrid triple quadrupole-ion trap mass spectrometer QTRAP 5,500 or 6,500 + (Sciex, Darmstadt, Germany) equipped with a Turbo-V-source operating in positive ESI mode. Analysis of the endocannabinoids was done using an Agilent 1290 Infinity I UHPLC system equipped with an Acquity UPLC BEH C18 UPLC column (100 Å ~ 2.1 mm, 1.7 μm, Waters, Eschborn, Germany). Quality control samples of three different concentration levels (low, middle, high) were run as initial and final samples of each run. The concentrations of the calibration standards, quality controls and samples were evaluated by Analyst software 1.6 and MultiQuant Software 3.0 (Sciex) using the internal standard method (isotope-dilution mass spectrometry). Calibration curves were calculated by linear or quadratic regression with 1/× weighting or 1/× 2 weighting. Variations in accuracy of the calibration standards were less than 15% over the range of calibration, except for the lower limit of quantification (LLOQ), where a variation in accuracy of 20% was accepted. For the acceptance of the analytical run, the accuracy of the QC samples had to be between 85 and 115% of the nominal concentration for at least 67% of all QC samples.

### Statistics

Group data are presented as mean ± SD or mean ± SEM, the latter for behavioral time courses as specified in the respective figure legends. Data were analyzed with SPSS 25, GraphPad Prism 8.4, Origin Pro 2020, and IntelliCage Plus (TSE systems) and FlowR (XBehavior)^84^ for IntelliCage experiments. Graphs were created in GraphPad Prism, SPSS, or FlowR; and they were arranged in Adobe Illustrator CC2020 and exported in TIFF format. Data were mostly normally distributed according to the Shapiro–Wilk-Test, unless stated otherwise. Groups were compared with unpaired, 2-sided Student's t tests. The Mann Whitney U test was used as a non-parametric alternative in case of violations of t test requirements. Time course data or multifactorial data were submitted to 2-way analysis of variance (ANOVA) using e.g. the factors 'time' and 'group'. In case of significant differences, groups were mutually compared at individual time points using post hoc t tests according to Dunnett, i.e. versus the control group, or according to Šidák. Posthoc comparisons for between subject factors (i.e. TBI versus sham) were not adjusted, if they were predefined by the experiment. In case of violations of sphericity, degrees of freedom were adjusted according to Huynh Feldt. Asterisks in figures show multiplicity-adjusted P values. The frequency distributions of nociceptive responses were fitted to a Gauss or Lorentzian normal distribution using least square regression. The goodness of fit was assessed via the sum-of-squares and QQ-plots. Linear regression analyses were used to assess the relationship of two parameters. A significant association required that the 95% confidence intervals of the slope of the regression line were different from zero.

In the IntelliCage, the number of visits and nosepokes were analyzed to assess overall activity, and the licks to assess drinking behavior and the success rate. Canonical Discriminant Analysis (CanDisc) was used to reveal the structure of behavioral patterns and assess the discrimination of the groups and prediction of group membership according to CanDisc scores. Eighteen independent behavioral parameters were used as loading. Cumulative correct visits of individual mice were plotted versus trials to assess the steepness of the learning curve. To assess the number of trials needed to achieve learning success, a probability test was used with the success criterion set to 0.35 for experiments with a random success of 0.25. Type 1 and type 2 errors were set to 0.05. Cosinor analysis of visiting frequencies and actograms were used to assess circadian rhythms. Mesor, acrophase and amplitude were subsequently compared between groups using unpaired, 2-sided t tests.

## Supplementary information

Supplementary file1

## Data Availability

No omics datasets were generated or analyzed during the current study.

## Abbreviations

CCI: Controlled cortical impact
TBI: Traumatic brain injury
eCB: Endocannabinoids
AEA: Anandamide
OEA: Oleoylethanolamide
PEA: Palmitoylethanolamide
1,2-AG, 1 & 2: Arachidonoylglycerol

## Behavior

FA: Free adaptation
NP: Nosepoke
PPL: Place preference learning
PAA: Place avoidance acquisition
PAEx: Place avoidance extinction
