# Supplementary material for: Low brain endocannabinoids associated with persistent non-goal directed nighttime hyperactivity after traumatic brain injury in mice
Source: Sci Rep. 2020 Sep 10;10:14929. doi: 10.1038/s41598-020-71879-x (PMC7483739; doi:10.1038/s41598-020-71879-x)
Supplement: Supplementary file 1 — Supplementary file1 [file 41598_2020_71879_MOESM1_ESM.pdf]

Low brain endocannabinoids associated with persistent non-goal directed nighttime hyperactivity after traumatic brain injury in mice

Alexandra Vogel[1], Annett Wilken-Schmitz[1], Regina Hummel[2], Manuel Lang[2], Robert Gurke[1], Yannick Schreiber[1], Michael K. E. Schäfer[2], Irmgard Tegeder[1]

Abstract

Traumatic brain injury (TBI) is a frequent cause of chronic headache, fatigue, insomnia, hyperactivity, memory deficits, irritability and posttraumatic stress disorder. Recent evidence suggests beneficial effects of pro-cannabinoid treatments. We assessed in mice levels of endocannabinoids in association with the occurrence and persistence of comparable sequelae after controlled cortical impact in mice using a set of long-term behavioral observations in IntelliCages, motor and nociception tests in two sequential cohorts of TBI/sham mice. TBI mice maintained lower body weights, and they had persistent low levels of brain ethanolamide endocannabinoids (eCBs: AEA, OEA, PEA) in perilesional and subcortical ipsilateral brain tissue (6 months), but rapidly recovered motor functions (within days), and average nociceptive responses were within normal limits, albeit with high variability, ranging from loss of thermal sensation to hypersensitivity. TBI mice showed persistent non-goal directed nighttime hyperactivity, i.e. they visited rewarding and non-rewarding operant corners with high frequency and random success. On successful visits, they made more licks than sham mice resulting in net over-licking. The lower the eCBs the stronger was the hyperactivity. In reward-based learning and reversal learning tasks, TBI mice were not inferior to sham mice, but avoidance memory was less stable. Hence, the major late behavioral TBI phenotype was non-goal directed nighttime hyperactivity and "over-licking" in association with low ipsilateral brain eCBs. The behavioral phenotype would agree with a "post-TBI hyperactivity disorder". The association with persistently low eCBs in perilesional and subcortical regions suggests that eCB deficiency contribute to the post-TBI psychopathology.

Supplementary Figure 1

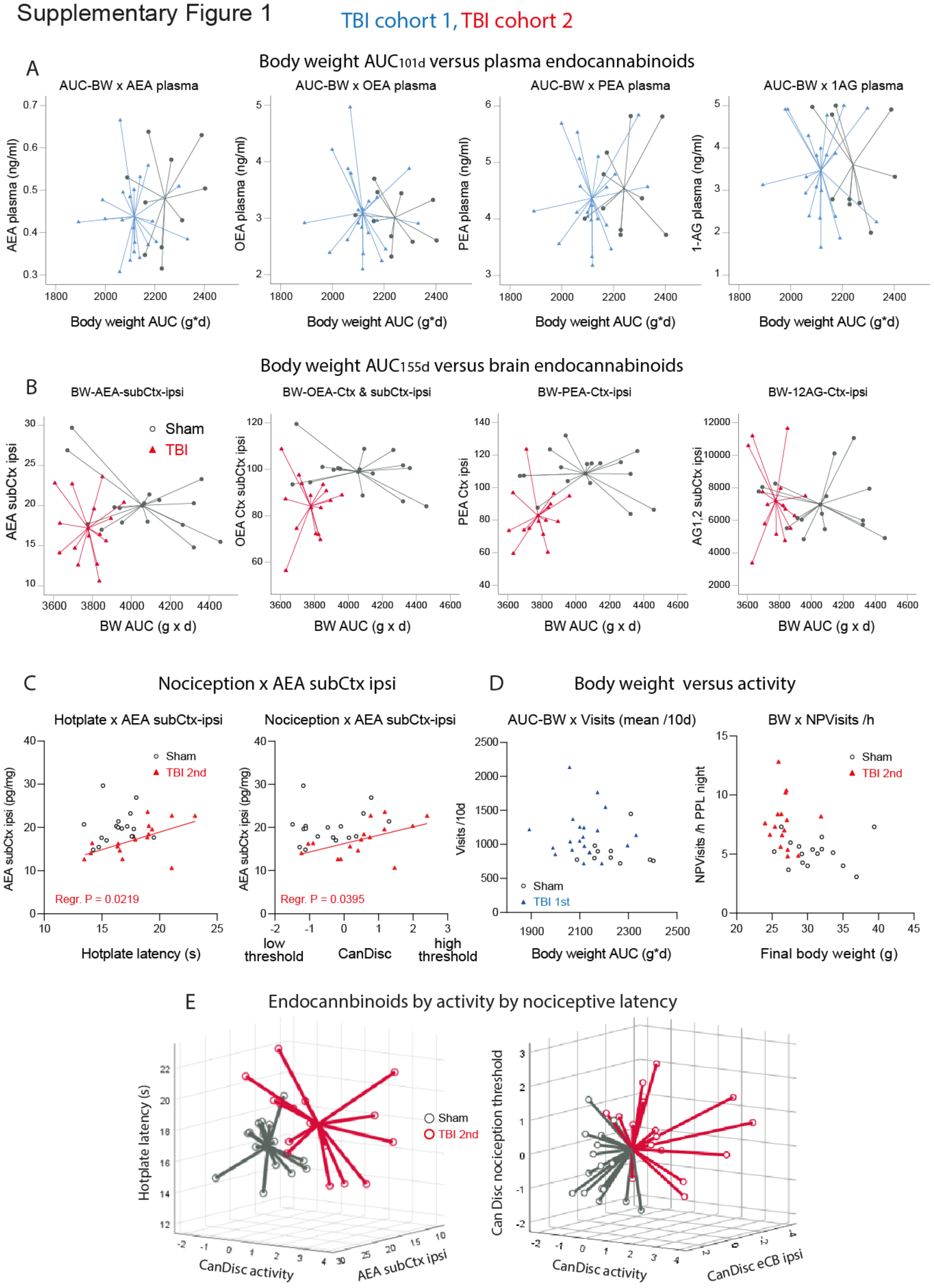

Supplementary Figure 1

Associations of body weights, IntelliCage activity, nociception and endocannabinoids

Results of TBI-cohort-1 are shown in blue, results of TBI-cohort-2 in red.

A: Plasma endocannabinoids versus body weight. Scatter plots with centroid spikes show body weight AUC's up to 101 days after TBI (cohort-1) versus plasma concentrations of endocannabinoids. Anandamide (AEA), oleoylethanolamide (OEA), palmitoylethanolamide (PEA) and 1-arachidonoylglycerol (1-AG). AUC_{101d} = area under the curve up to 101 days. Each dot is a mouse.

B: Brain endocannabinoids versus body weight. In analogy to A, the scatter plots with centroid spikes show body weight AUCs after TBI (cohort-2) versus ipsilateral brain concentrations of endocannabinoids in ipsilateral brain (shown in Fig. 1). Ctx = cortex perilesional, subCtx = subcortical underneath the lesion. AUC_{155d} = AUC up to 155 days after TBI.

C: Anandamide versus nociceptive sensitivity. Linear association of nociceptive hotplate latencies 5-6 months after TBI (close to tissue sampling) versus AEA concentrations in ipsilateral subcortical brain (subCtx ipsi) (cohort-2), and linear association of canonical discriminant (CanDisc) scores for nociceptive thresholds (hotplate, dynamic von Frey, Hargreaves latencies as input) versus AEA. Low scores indicate low thresholds (i.e. high sensitivity). The linear regression lines are shown for TBI mice, where the slopes differed significantly from "zero" (n.s. for sham).

D: Body weight versus activity. Scatter plots of the body weight AUC (area under the time curve up to 101 dafter TBI) versus the individuals' average visit counts per 10 day-periods (cohort-1) and corresponding plots for cohort-2. "NPVisits /h" show visits with nosepokes but without licks per hour during place preference learning in the night.

E: 3D scatter plots with centroid spikes of **AEA** (subCtx ipsilateral) versus **Hotplate latencies** versus **activity scores** (CanDisc with Visits and Licks as input) and 3D scatter plots of **CanDisc scores** with spikes arising from the origin. Group membership prediction was 88.2% for sham and 93.8% for TBI mice.

Spiked scatter plots in A, B and E were created in SPSS 25 (https://www.ibm.com/de) and exported as enhanced metafile (emf). Graphs in C and D were created in Graphpad Prism 8.4 (https://www.graphpad.com) and exported as emf. Graphs were arranged and labeled in Adobe Illustrator CC2020 (https://www.adobe.com/de), and exported to TIFF format.

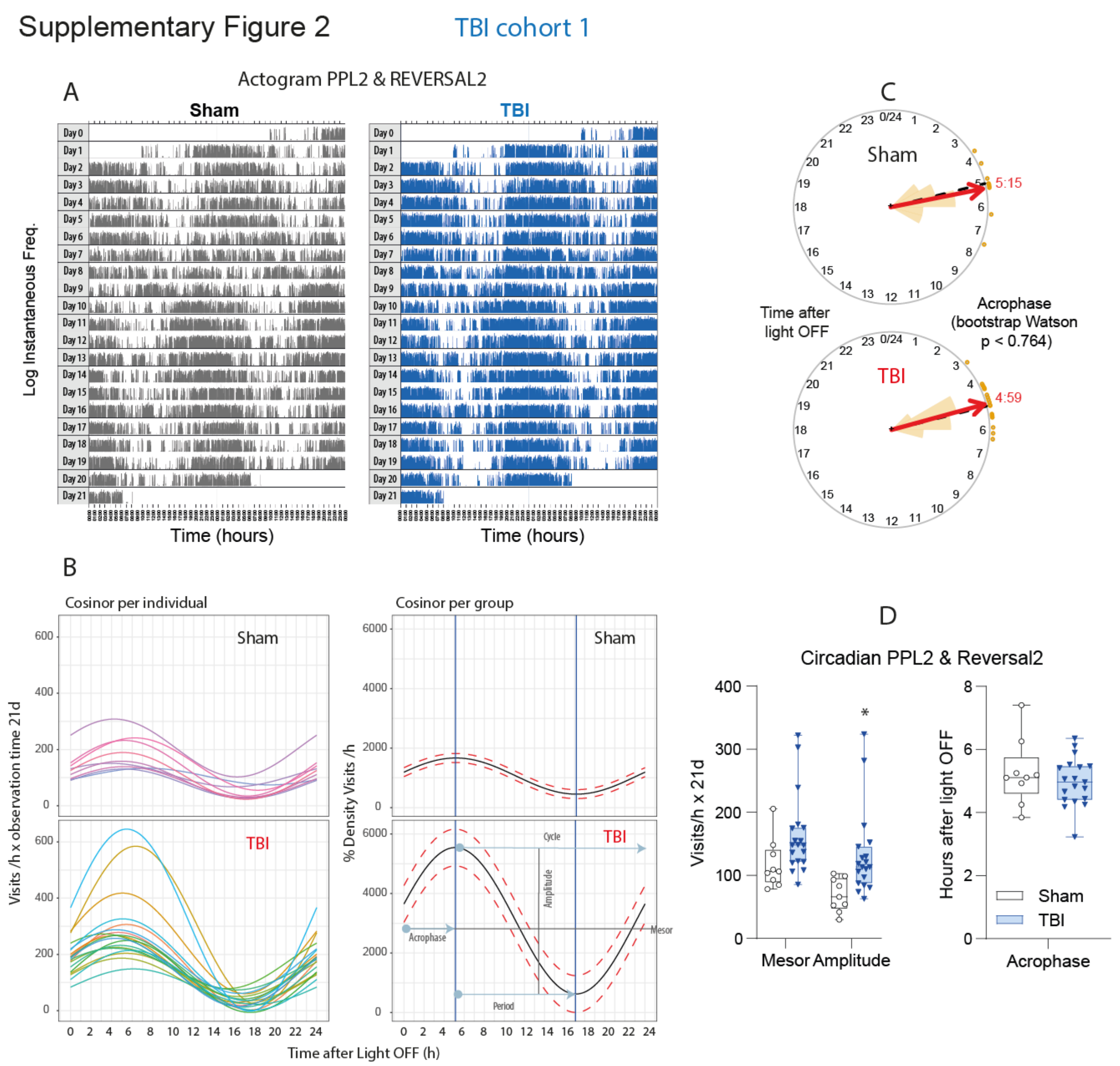

Supplementary Figure 2

Analysis of circadian rhythms in TBI/sham mice of cohort-1 in analogy to circadian analysis of cohort-2 in Figure 5.

A: Exemplary actogram showing the circadian rhythms of corner visiting activity in TBI/sham mice of cohort 1 during the second place preference learning and reversal learning tasks (PPL2/REV2). The Y-axes show the logarithms of the instantaneous frequency, which is the reciprocal of the time from start of one visit to start of next visit.

B: Cosinor analysis of visiting activity of individual mice and the groups. The circadian parameters are shown in the bottom right graph. The red dotted lines show the 95% CI.

C: Circular presentation of the acrophases i.e. the time from Light OFF to maximum activity.

D: Quantitative and statistical comparison of major circadian parameters, acrophase, amplitude and mesor, as explained in B. The box shows the interquartile range, the line is the median, the whiskers show minimum to maximum, dots are individual mice. The asterisk indicates significant differences between groups; 2-sided, unpaired T-tests for each parameter, *P <0.05, n=9 sham (initially 10, one drop out), n=19 TBI (initially 21, two drop outs).

Circadian data were analyzed with FlowR (XBehavior; http://www.xbehavior.com). Images in A, B, C were exported from FlowR as .png, and actograms in A were colored to fit the groups (grey and blue)

in Adobe Photoshop CC2020. Data were exported as tab-separated txt files, imported in Microsoft Excel 2016, and Graphs in D were created with Graphpad Prism 8.4 (https://www.graphpad.com) and exported as emf. Graphs were arranged and labeled in Adobe Illustrator CC2020 (https://www.adobe.com/de), and exported to TIFF format.
